# APC controls Wnt-induced β-catenin destruction complex recruitment in human colonocytes

**DOI:** 10.1038/s41598-020-59899-z

**Published:** 2020-02-19

**Authors:** Taybor W. Parker, Kristi L. Neufeld

**Affiliations:** Department of Molecular Biosciences, University of Kansas, Lawrence, KS USA

**Keywords:** Colon cancer, Extracellular signalling molecules

## Abstract

Wnt/β-catenin signaling is essential for intestinal homeostasis and is aberrantly activated in most colorectal cancers (CRC) through mutation of the tumor suppressor *Adenomatous Polyposis Coli* (*APC*). APC is an essential component of a cytoplasmic protein complex that targets β-catenin for destruction. Following Wnt ligand presentation, this complex is inhibited. However, a role for APC in this inhibition has not been shown. Here, we utilized Wnt3a-beads to locally activate Wnt co-receptors. In response, the endogenous β-catenin destruction complex reoriented toward the local Wnt cue in CRC cells with full-length APC, but not if APC was truncated or depleted. Non-transformed human colon epithelial cells displayed similar Wnt-induced destruction complex localization which appeared to be dependent on APC and less so on Axin. Our results expand the current model of Wnt/β-catenin signaling such that in response to Wnt, the β-catenin destruction complex: (1) maintains composition and binding to β-catenin, (2) moves toward the plasma membrane, and (3) requires full-length APC for this relocalization.

## Introduction

**Figure 1:**
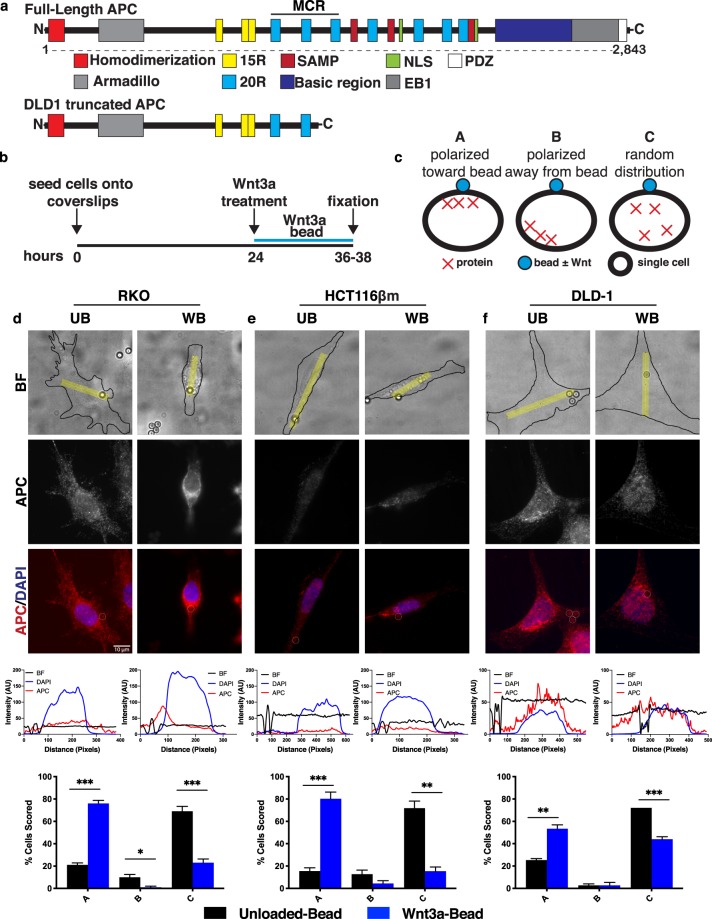
APC orientation in response to localized Wnt-3a in a panel of colorectal cancer cell lines. (**a**) Full-length APC and its interaction domains. Truncated APC found in DLD1 cells results in loss of the C-terminal half of the protein. (**b**) Schematic of experimental design. One day after seeding onto fibronectin-coated coverslips, cells were treated with Wnt3a-beads for 12–14 hours, then fixed and processed for immunofluorescence microscopy. (**c**) Scoring criteria for protein localization. (**d**) Representative images of APC localization in RKO cells treated with Unloaded-bead (UB) or Wnt3a-bead (WB). Representative line scan of bright-field (BF, yellow region analyzed) demonstrates increased APC signal intensity near the WB but not the UB. For line scan graphs, intensity in arbitrary units (AU) of bead = black; DAPI = blue; APC = red. Below line scan graphs, results from three independent experiments were averaged and graphed with error bars representing SEM. For each experiment 25 cells were scored per condition. Statistical analysis by t-test: *P < 0.05; **P < 0.01; ***P < 0.001. (**e,f**) Representative images, line scans, and scoring results for HCT116βm and DLD-1 cells, respectively. Scale bar, 10 μm.

The Wnt signaling pathway is essential for cell proliferation, cell polarity, developmental cell-fate determination, and tissue homeostasis^1^. As a result, deregulation of Wnt signaling is often associated with cancer and other diseases^2,3^. Notably, over 90% of colorectal cancers (CRC) have mutations that activate the Wnt pathway with over 80% containing mutations in the Wnt antagonist *Adenomatous Polyposis Coli* (*APC*)^4^. Human *APC* mutations commonly arise in a central region of the open reading frame, referred to as the “mutation cluster region” (MCR, Fig. 1a), resulting in a truncated protein product^5^. APC truncation results in loss of multiple β-catenin binding sites (20R), Axin interaction sites (SAMP), nuclear localization sequences, and a C-terminal basic region which mediates cytoskeletal interactions (Fig. 1a). Germline or sporadic *APC* mutations in colon stem cells lead to polyp formation and are considered initiating events in colorectal tumorigenesis^6,7^. In the context of Wnt signaling, it is well established that APC acts as a scaffold in the β-catenin destruction complex. Given that other proteins involved in this complex could be altered to activate Wnt signaling, it is curious that *APC* mutations predominate in CRCs. One potential explanation for this is that APC performs additional critical functions both inside and outside the context of Wnt signaling.

The canonical Wnt pathway serves to regulate the level of transcriptional coactivator β-catenin^8,9^. To prevent aberrant transcriptional activation in the absence of Wnt, a cytoplasmic β-catenin destruction complex composed of APC, Axin, CK1-α, and GSK-3β maintains low levels of β-catenin through sequestration, phosphorylation, and β-TrCP-mediated ubiquitination, leading to proteasomal degradation^10–12^. However, if a Wnt ligand binds the co-receptors Frizzled (FZD) and low-density lipoprotein receptor 5/6 (LRP5/6) to form the heterotrimeric “signalosome,” the β-catenin destruction complex is inhibited through an incompletely resolved mechanism^13,14^. While the downstream effects of Wnt signaling have been extensively analyzed, the specific molecular events which cause destruction complex inhibition are not well understood. Further, the exact roles of APC in normal destruction complex function have remained elusive, and it has not been determined whether APC is also involved in promoting the transduction of a Wnt signal.

Wnt ligands are required for intestinal stem cell (ISC) self-renewal and crypt homeostasis^15–17^. Traditionally, these intestinal Wnt ligands have been thought to diffuse from the crypt base toward the luminal surface to form a signaling gradient. However, Wnt was recently shown to predominantly engage with FZD receptors on the ISCs that were immediately adjacent to Wnt-secreting cells and not those that were further removed from the Wnt source. These findings are inconsistent with a Wnt diffusion model, but instead support a model whereby Wnt is initially bound to receptors following secretion and forms a gradient through receptor turnover and cell division^18^. Therefore, intestinal Wnt signaling may convey positional information within the crypt and direct intracellular protein localization based on the location of the Wnt source. In the current study, we will examine the effect of a local, immobilized Wnt signal on colon epithelial cells.

Several challenges have historically limited our understanding of Wnt signaling dynamics as it relates to intestinal homeostasis in the normal and cancerous state. Due to the widespread use of the *Apc*^*Min/+*^ mouse model, much focus has been directed to the small intestine rather than the colon, the site of most tumor-initiating *APC* mutations in humans^19^. Further, very little is known about Wnt signaling in colon epithelial cells that are from non-malignant origin, as most studies utilize only cultured CRC cell lines. Finally, prior research has mostly relied on overexpression of specific Wnt pathway components or cells treated with soluble Wnt in the media, limiting the ability to elucidate endogenous β-catenin destruction complex dynamics in response to a local Wnt signal (reviewed in^3^).

Here, we examine the response of endogenous β-catenin destruction complex components to a locally presented Wnt signal in human colon epithelial cells of both malignant and non-malignant origin. We demonstrate for the first time that a localized Wnt source can recruit the signalosome and β-catenin destruction complex in colon epithelial cells and find that this Wnt-induced recruitment requires full-length APC. Our work identifies a novel role for APC in the regulation of destruction complex movement toward the membrane following Wnt exposure.

## Results

### Wild-type APC, but not truncated mutant APC is recruited toward local Wnt3a in human colon cancer cells

Since *APC* is mutated in >80% of colorectal cancers and is a major scaffolding protein for the β-catenin destruction complex, we first asked whether APC redistributes toward a localized Wnt3a signal in a panel of human CRC cell lines. Three colon cancer cell lines, each with a different Wnt pathway status, were used: RKO (intact Wnt signaling pathway), HCT116βm (WT APC, stabilized β-catenin due to Ser45 deletion)^20^, and DLD1 cells (APC truncation at amino acid 1452, WT β-catenin, see Fig. 1a). Previous studies relied on Wnt addition to cell culture media, thus limiting the ability to examine responses to a localized stimulus. To address this issue and examine endogenous protein response to a locally applied Wnt source – we treated cells with Wnt3a-conjugated or Unloaded-beads.

Cells treated for 12–14 hours with Wnt- or Unloaded-beads were fixed and stained for APC (Fig. 1b). Protein localization was scored as: (A) toward the bead, (B) away from the bead, or (C) no obvious protein polarization (Fig. 1c). Scoring results were validated with line-scan analysis. Full-length APC localized toward the Wnt3a-bead in 76% of RKO (Fig. 1d) and 80% of HCT116βm cells (Fig. 1e). In contrast, truncated APC localized toward the Wnt-bead in only 53% of DLD-1 cells (Fig. 1f). In all cases, the majority of cells treated with Unloaded-beads displayed random distribution of APC, suggesting that physical contact with an Unloaded-bead is not sufficient to re-orient APC. These data demonstrate that Wnt exposure induces APC re-localization toward the Wnt source in multiple human CRC lines. Furthermore, this process appears to be compromised in cells carrying truncated APC but remains functional in cells with an activated Wnt/β-catenin transcriptional program due to stabilized β-catenin. The phenotypic consequences of truncated APC have been previously postulated to act in a “just-right” signaling model, in which truncated APC retains partial β-catenin regulatory function to allow a specific level of Wnt activation^21^. Because truncated APC shows reduced ability to localize toward a Wnt source, it is possible that this partial localization is a method to obtain a precise level of Wnt-response.

### Wnt components are recruited toward localized Wnt3a in cells with an intact Wnt signaling pathway

Since APC is a key scaffold for the β-catenin destruction complex, we reasoned that additional members of the destruction complex and signalosome may also localize near a Wnt-bead. RKO cells with an intact Wnt signaling pathway were treated with beads and then stained for the scaffolding protein Axin1, kinases CK1-α and GSK-3β, the Wnt receptor FZD7, the ubiquitin ligase β-TrCP, and β-catenin. FZD7 was chosen for its defined role in gastrointestinal homeostasis^22–24^.

**Figure 2:**
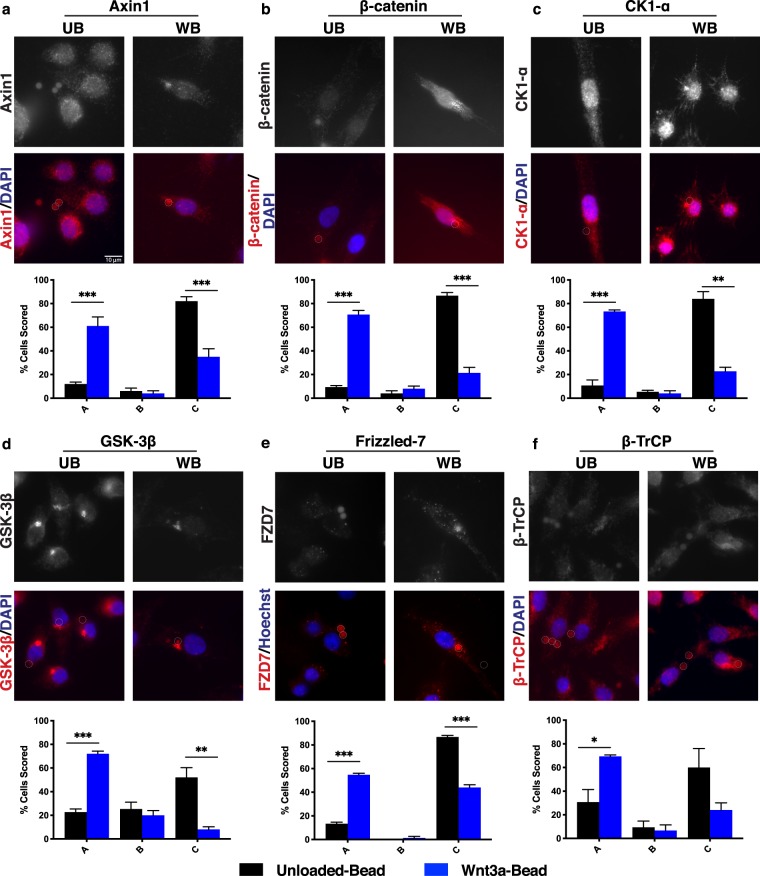
Wnt signaling pathway components localize toward Wnt-bead. Representative images of RKO cells (CRC line with intact Wnt signaling pathway) treated with UB or WB as in Fig. 1a and stained for (**a**) Axin1, (**b**) β-catenin, (**c**) CK1-α, (**d**) GSK-3β, (**e**) Frizzled-7, or (**f**) β-TrCP. Below representative images are bar graphs of the scoring quantification. Data averaged from three independent experiments (n = 25 cells per condition). Error bars, SEM; Statistical analysis by t-test: *P < 0.05; **P < 0.01; ***P < 0.001. Representative line scans provided in supplemental Fig. 1. Scale bar, 10 μm.

Distinct Axin1 puncta were observed near the Wnt-bead (Fig. 2a). While RKO cells contain low levels of β-catenin due to an intact Wnt pathway, they do have visible β-catenin staining which can be scored. Treatment with Unloaded-beads failed to induce relocalization of β-catenin (Fig. 2b). However, in cells treated with a Wnt-bead, β-catenin levels increase and β-catenin localized toward the bead (Fig. 2b). Of note, the ubiquitin ligase β-TrCP also localized toward the bead (Fig. 2f). These findings suggest that upon Wnt/FZD binding, the cytoplasmic destruction complex relocates to the membrane and is able to recruit β-TrCP but falls short of targeting β-catenin for destruction. This result is consistent with a model proposed by Li *et al*. whereby the destruction complex saturates with β-catenin, leaving newly synthesized β-catenin to control downstream Wnt target gene expression following Wnt stimulation^25^. The kinases CK1-α and GSK-3β were also detected near the Wnt-bead (Fig. 2c,d), as was the Wnt receptor FZD7 (Fig. 2e).

**Figure 3:**
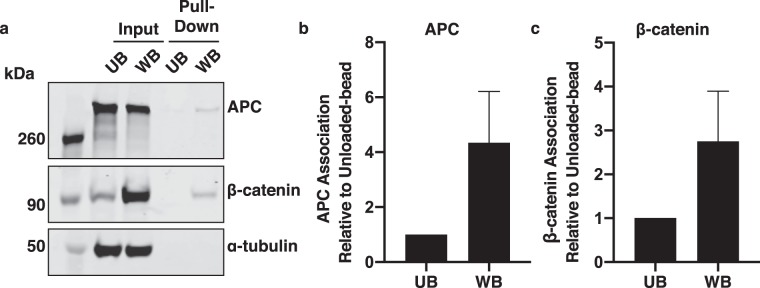
Wnt-beads pull-down APC and β-catenin. (**a**) RKO cells treated with Unloaded-beads or Wnt-beads were lysed and proteins pulled-down with the beads. APC and β-catenin were detected in the Wnt-bead pull-down but not the Unloaded-bead pull-down. Images are from the same gel/membrane cut between 50 and 70 kDa and probed for APC and β-catenin (above 70 kDa) or α-tubulin (below 50 kDa). Multi-channel imaging was performed for APC and β-catenin through IRDye infrared secondary antibodies to allow detection on the same membrane. (**b**,**c**) Quantification of APC and β-catenin protein pulled-down by Wnt-beads compared to Unloaded-beads from five independent experiments. Protein levels of APC and β-catenin that were pulled down by Unloaded-beads or Wnt-beads were divided by the respective input protein levels and normalized to the Unloaded-bead to demonstrate fold change of APC (**b**) and β-catenin (**c**). Error bars, SEM.

To test for a physical interaction between the Wnt-coated beads and destruction complex components, the beads were used to “pull down” Wnt and associated proteins from lysates of Wnt-bead-treated cells. APC and β-catenin each associated more with Wnt-beads than with Unloaded-beads (Fig. 3). Together, these data demonstrate that signalosome and destruction complex proteins concentrate near a local Wnt3a cue and do not fully disassemble in response to ~12 hour exposure to a Wnt3a bead. Furthermore, these data support a model in which β-catenin remains associated with the destruction complex following Wnt stimulation.

### Stabilized β-catenin does not impact Wnt component localization in response to Wnt3a

In colorectal cancer, mutations in the APC and β-catenin encoding genes appear to be mutually exclusive. Though most CRCs have *APC* mutations, about half of the small subset of colorectal cancers with wild-type *APC* harbor mutations that activate β-catenin^26^. These mutations typically result in loss of specific β-catenin residues whose phosphorylation is critical for ubiquitin conjugation and proteasome-mediated destruction. To determine whether such “stabilizing” β-catenin mutations would compromise orientation of Wnt signaling components toward a localized Wnt3a, we examined HCT116βm cells. Unlike the parental HCT116 cells, which express both wild-type and mutant β-catenin, HCT116βm possess only one mutant β-catenin allele encoding a Ser45 deletion^20^. Ser45 phosphorylation by CK1-α primes the successive phosphorylation of Thr41, Ser37, and Ser33 by GSK-3β, a prerequisite for recognition and ubiquitination by β-TrCP^10–12^. Note that APC localized toward the Wnt-bead in both RKO and HCT116βm cells (Fig. 1d,e).

**Figure 4:**
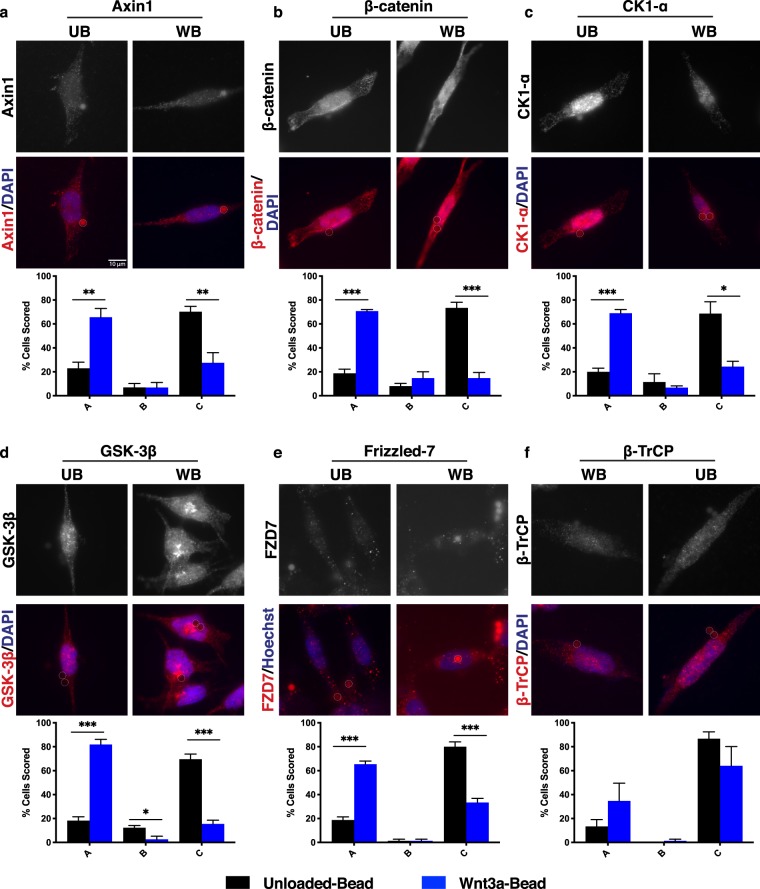
Activated Wnt signaling through stabilized β-catenin does not alter Wnt-mediated signaling pathway component localization. Representative images of HCT116βm cells (CRC line with single allele encoding β-catenin that cannot be phosphorylated by CK1-α) treated with UB or WB as in Fig. 1a. Phosphorylation by CK1-α is reported to be a prerequisite for phosphorylation by GSK-3β and subsequent ubiquitin-mediated degradation. Representative images and scoring shown for (**a**) Axin1, (**b**) β-catenin, (**c**) CK1-α, (**d**) GSK-3β, (**e**) Frizzled-7, and (**f**) β-TrCP. Data averaged from three independent experiments (n = 25 cells per condition). Error bars, SEM; Statistical analysis by t-test: *P < 0.05; **P < 0.01; ***P < 0.001. Representative line scans provided in supplemental Fig. 2. Scale bar, 10 μm.

As seen in RKO cells, Axin1 localized in distinct puncta near the Wnt-bead in HCT116βm cells (Fig. 4a). Despite expressing only stabilized β-catenin, HCT116βm cells exhibited both CK1-α and GSK-3β localized toward the Wnt-bead (Fig. 4c,d). This finding demonstrates that CK1-α recruitment to the destruction complex is not dependent on Ser45 of β-catenin, nor is GSK-3β recruitment dependent on Ser45 phosphorylation. FZD7 was also detected near the Wnt-bead (Fig. 4e). Notably, β-catenin also localized near the Wnt-bead (Fig. 4b), suggesting that the destruction complex maintains association with stabilized β-catenin, even in the absence of degradation. On the other hand, β-TrCP failed to localize toward the Wnt-bead in HCT116βm cells (Fig. 4f), demonstrating that β-catenin Ser45 is necessary for β-TrCP association with the destruction complex. Therefore, β-TrCP does not appear to be an inherent member of the destruction complex, but rather, is recruited following β-catenin phosphorylation. Together, these results demonstrate that an activated Wnt pathway through β-catenin stabilization is not sufficient to block orientation of core destruction complex proteins toward localized Wnt. β-TrCP failed to localize toward the Wnt cue, consistent with CK1-α phosphorylation of β-catenin as a prerequisite for β-TrCP recruitment.

### Cells lacking full-length APC are compromised for Wnt component localization toward Wnt

Both HCT116βm and DLD1 cells possess an activated Wnt/β-catenin transcriptional program, induced by mutation of β*-catenin* or *APC*, respectively. As described, with the exception of β-TrCP, Wnt pathway components remained able to orient toward a localized Wnt source in HCT116βm cells (Fig. 4). DLD1 cells have a mutation in the mutation cluster region that leads to a truncated APC protein lacking the Axin-binding SAMP motifs and most of the β-catenin-binding 20aa repeats (Fig. 1a). However, this truncated APC was reported to co-precipitate with Axin1, suggesting that it maintains its scaffolding properties^25^. Given that truncated APC appeared compromised for orientation toward a Wnt-bead in DLD1 cells (Fig. 1f), we asked whether other β-catenin destruction complex members also displayed diminished localization toward a Wnt3a source.

**Figure 5:**
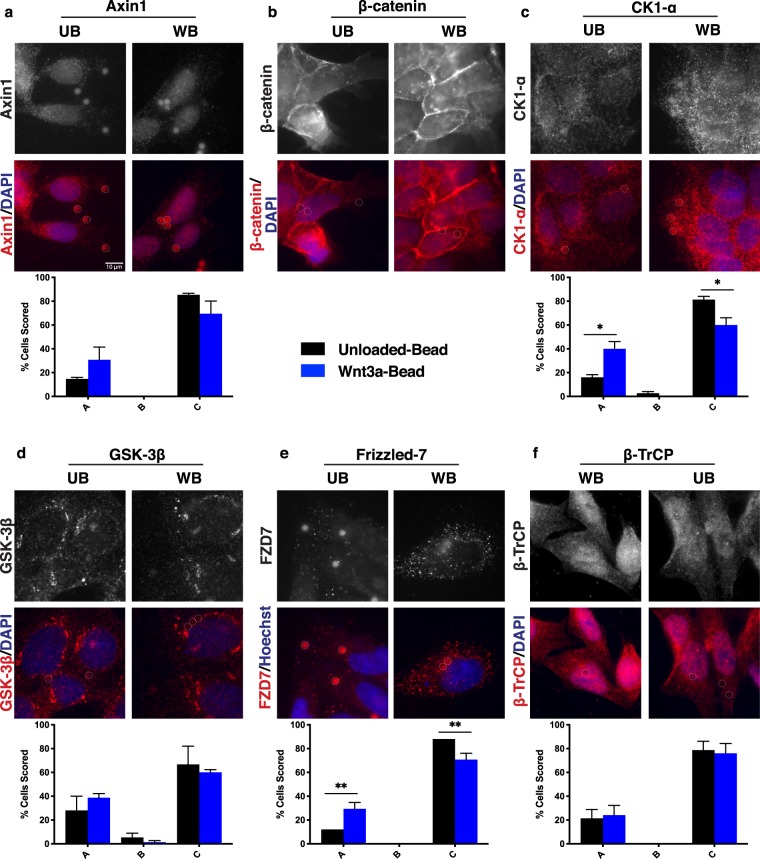
CRC cells with truncated APC display diminished association of Wnt signaling pathway components toward localized Wnt. Representative images of DLD-1 cells (CRC line a expressing only truncated APC (amino acids 1–1472)) treated with UB or WB. Representative images and scoring shown for (**a**) Axin1, (**b**) β-catenin, (**c**) CK1-α, (**d**) GSK-3β, (**e**) Frizzled-7, and (**f**) β-TrCP. Data averaged from three independent experiments (n = 25 cells per condition). Error bars, SEM; Statistical analysis by t-test: *P < 0.05; **P < 0.01; ***P < 0.001. Representative line scans provided in supplemental Fig. 3. Scale bar, 10 μm.

If APC only serves as a destruction complex scaffold, then we would expect normal destruction complex orientation in DLD1 cells. Instead, we found that Axin1 localization to the Wnt3a-bead was detected in only 30% of DLD-1 cells (Fig. 5a), compared to 61% of RKO cells (Fig. 2a) and was not significantly different than localization to the Unloaded-bead. GSK-3β and β-TrCP were also compromised for Wnt-bead orientation in DLD1 cells (Fig. 5d,f). Of all proteins analyzed in DLD1 cells, only CK1-α and FZD7 were localized toward the Wnt-bead more than the Unloaded-bead (Fig. 5c,e). However, even this Wnt-bead localization was observed much less frequently in DLD1 cells than in RKO or HCT116βm cells, which contain full-length APC. Subcellular β-catenin reorientation could not be assessed by visual inspection of DLD1 cells due to overall high protein levels. However, line scan analyses demonstrated that β-catenin also failed to relocate in response to a Wnt-bead (Fig. S3).

Together, these results indicate that full-length APC is necessary for optimal destruction complex orientation in response to a Wnt cue. Given that truncated APC co-precipitates with Axin1 and the destruction complex in DLD1 cells^25^, our results also suggest that APC mediates destruction complex localization by a mechanism independent of, or in addition to, its role as a scaffold for the complex. Truncated APC in DLD1 cells lacks domains that bind dynein^27^, kinesin^28^, and microtubules^27–30^. These interactions potentially contribute to movement of the destruction complex toward the Wnt source. However, because localization of FZD7 and CK1-α toward the Wnt-bead was decreased, but still present, it appears that truncated APC retains some ability to localize the complex or that alternative mechanisms may be able to localize these proteins in the absence of full-length APC.

### APC loss impairs Wnt-induced destruction complex reorientation

**Figure 6:**
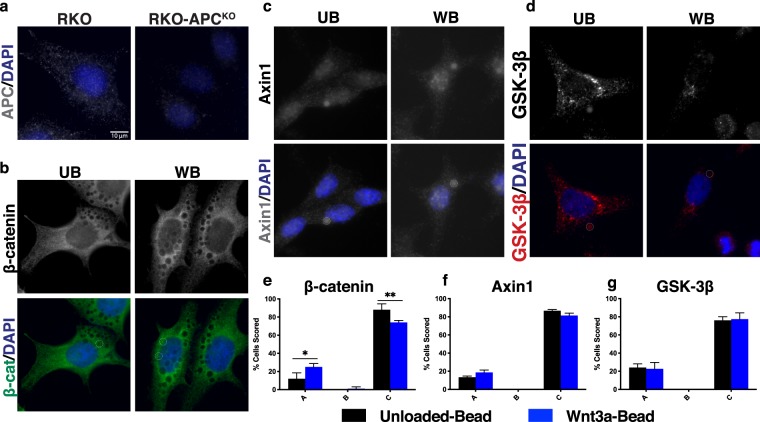
APC knockout in CRC cells impairs Wnt-induced β-catenin destruction complex localization. (**a**) APC staining in RKO and RKO-APC^KO^ cells. (**b**–**d**) Representative images of RKO-APC^KO^ cells treated with UB or WB. Representative images and scoring shown for (**b**,**e**) β-catenin, (**c**,**f**) Axin1, and (**d**,**g**) GSK-3β. Data averaged from three independent experiments (n = 25 cells per condition). Error bars, SEM; Statistical analysis by t-test: *P < 0.05; **P < 0.01; ***P < 0.001. Scale bar, 10 μm.

Given that the β-catenin destruction complex localized toward a Wnt-bead in CRC cell lines with wild-type APC but was impaired in cells with truncated APC, we hypothesized that destruction complex localization requires full-length APC. To test the APC-dependence of Wnt-induced destruction complex localization, we utilized RKO-APC^KO^ cells, generated through CRISPR/Cas9 by Lee and colleagues^31^. Immunofluorescent microscopy revealed reduced APC signal in RKO-APC^KO^ cells compared to the parental RKO cell line (Fig. 6a). While parental RKO cells displayed Wnt-induced localization of β-catenin in 70% of cells scored (Fig. 2b), this localization was seen in only 25% of the RKO-APC^KO^ cells (Fig. 6b,e). Further, Axin1 and GSK-3β failed to relocalize toward a Wnt-bead in the RKO-APC^KO^ cells (Fig. 6c,f,d,g). These data demonstrate that Wnt-induced destruction complex localization in CRC cells is dependent on APC.

### Wnt triggers destruction complex reorientation in normal human colonic epithelial cells in an APC-dependent manner

In normal colon tissue, adult stem cells facilitate the continual regeneration of the epithelial lining and rely on Wnt ligand for maintenance of the stem cell niche. Having observed Wnt-mediated re-localization of the β-catenin destruction complex in human colon cancer cell lines, we wondered if normal human colon stem cells also displayed this property and if so, whether this relocalization was APC-dependent. To address this, we sought human colonic epithelial cells (HCECs) that were isolated from normal tissue and propagated in culture under near physiological conditions (low oxygen and sera). Shay and colleagues isolated HCECs from routine colonoscopy and immortalized them with expression of cyclin-dependent kinase 4 (Cdk4) and human telomerase (hTERT)^32^. These “HCEC 1CT” cells also express endogenous stem cell markers and are able to differentiate into multiple cell lineages making them a unique and valuable model of normal colon epithelial stem cells.

**Figure 7:**
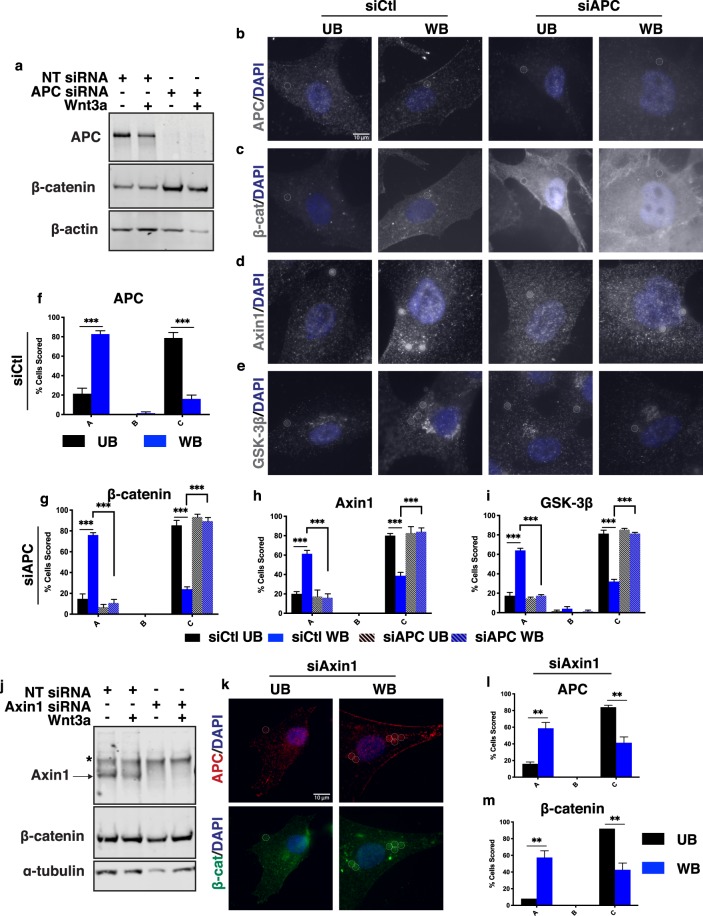
The β-catenin destruction complex localizes toward Wnt in normal human colonic epithelial cells in an APC-dependent manner. Representative images and scoring analysis of HCEC 1CT cells (immortalized cells from normal human colon) treated with control siRNA (siCtl), APC siRNA (siAPC), or Axin1 siRNA (siAxin1) for 48 hours prior to UB or WB treatment. (**a**) APC knock-down in HCEC 1CT cells. Images are from the same gel/membrane. Membrane was cut between 50 and 70 kDa and probed for APC and β-catenin (above 70 kDa) or β-actin (below 50 kDa). Multi-channel imaging was performed for APC and β-catenin through IRDye infrared secondary antibodies so that they could be probed on the same membrane. (**b,c,f,g**) HCEC 1CT stained for β-catenin and APC (channels separated). (**d,h**) HCEC 1CT stained for Axin1. (**e,i**) HCEC 1CT stained for GSK-3β. (**j**) Axin1 knock-down in HCEC 1CT cells. Images are from the same gel/membrane cut as in (**a**) and probed for Axin1 and β-catenin (above 70 kDa) or α-tubulin (below 50 kDa). Asterisk indicates non-specific band. (**k**–**m**) Representative images and scoring analysis of APC and β-catenin localization in Axin1-depleted HCEC 1CT cells. Data averaged from three independent experiments (n = 25 cells per condition). Error bars, SEM: Statistical analysis by t-test: *P < 0.05; **P < 0.01; ***P < 0.001. Scale bar, 10 μm.

Using HCEC 1CT cells, we depleted APC levels with siRNA (siAPC) and then determined localization of β-catenin, Axin1 and GSK3β. APC knock-down was efficient in HCEC 1CT and resulted in increased β-catenin protein levels, as expected (Fig. 7a). In cells treated with scrambled siRNA (siCtl), Wnt-bead exposure resulted in re-orientation of APC, β-catenin, Axin1, and GSK-3β toward the Wnt source (Fig. 7b–i). Therefore, normal non-transformed human colonic epithelial cells maintain the ability to re-orient the β-catenin destruction complex toward a Wnt cue. Remarkably, APC knock-down resulted in complete loss of β-catenin, Axin1, and GSK-3β orientation toward the Wnt-bead (Fig. 7c–e,g–i). Distinct β-catenin puncta were visualized near Wnt-beads in siCtl HCEC 1CT cells. In contrast, β-catenin levels were increased with diffuse cytoplasmic staining and prominent nuclear localization in HCEC 1CT cells depleted of APC but lacked localization toward the Wnt-bead (siAPC, Fig. 7c). These data are in agreement with the findings in RKO-APC^KO^ cells (Fig. 6).

Like APC, Axin1 is a key scaffolding protein in the β-catenin destruction complex, and thus, might control proper localization of the complex. Using HCEC 1CT cells, we depleted Axin1 with siRNA (siAxin1) and determined the localization of APC and β-catenin. Both APC and β-catenin still localized toward the Wnt-bead in Axin1-depleted HCEC 1CT cells (Fig. 7l,m). However, this bead-induced redistribution did appear to be slightly compromised compared to that seen in siCtl HCEC 1CT cells. Given that APC-depletion resulted in complete loss of localization of destruction complex components, we conclude that APC is necessary and sufficient for Wnt-induced localization of the destruction complex, while Axin1 can enhance the localization, but is expendable.

Together, these data demonstrate that the β-catenin destruction complex retains the ability to orient toward a localized Wnt source in HCEC 1CT cells and reveal a requirement and novel function of full-length APC in destruction complex trafficking toward a Wnt cue.

## Discussion

To investigate the role of APC in Wnt-directed destruction complex localization, we first explored signalosome and destruction complex response in commonly used CRC cell lines with varying Wnt pathway status. The data from these experiments provide novel findings that the β-catenin destruction complex re-localizes toward Wnt in colon epithelial cells and that this redistribution is impaired in DLD1 cells expressing a truncated form of APC. Truncated APC is present in the majority of human CRC, and results in loss of the C-terminal half of APC, which contains domains involved in destruction complex assembly, nuclear localization, and cytoskeletal interactions. Further, we demonstrate that APC loss ablates Wnt-induced destruction complex localization. Unlike the parental RKO cell line, Axin1, GSK-3β and β-catenin are unable to localize toward a Wnt-bead in RKO-APC^KO^ cells. We extended this study to a non-transformed human colon epithelial cell line which exhibits stem cell characteristics and found that Wnt-directed destruction complex localization also occurs in colon epithelial cells of non-malignant origin. When compared to the protein distribution in DLD1 cells, which were already limited in signalosome and destruction complex reorientation in response to localized Wnt, APC depletion in the non-transformed human colon epithelial cells completely abolished Wnt-induced destruction complex localization. Combined with a previous report that the truncated APC found in DLD1 remains associated with destruction complex protein Axin1^25^, it appears that APC utilizes its function in scaffolding as well as interactions in the C-terminus to fully traffic the destruction complex toward a Wnt cue.

Numerous models of the events following Wnt receptor activation have been proposed, however the specific molecular proceedings that occur are still debated due to the use of overexpression of Wnt pathway components, global addition of Wnt to culture media, or the wide range of cell and tissue types used. Many models propose that the destruction complex is either inactivated or partially disassembled following Wnt/co-receptor interaction^3,33–36^. However, other studies have demonstrated that some of the destruction complex may be targeted to the plasma membrane following receptor activation^25,37,38^. Recently, an elegant study exposed mouse embryonic stem cells to Wnt3a-conjugated beads, resulting in APC, β-catenin, and LRP6 localization toward a Wnt-bead and also found that the local Wnt cue could trigger asymmetric cell division^39^. To our knowledge, this is the only study examining the orientation of signalosome and destruction complex components in response to localized Wnt and was performed in pluripotent cells of nonhuman origin and limited to three endogenous proteins.

Our study builds upon the current model of Wnt signal transduction and brings to light a new role for APC. Here, we report the requirement of APC for proper Wnt-induced localization of the β-catenin destruction complex in both malignant CRC cell lines and in non-transformed colon epithelial cells. In contrast to our findings, others have demonstrated that key members of the complex are degraded or endocytosed following prolonged Wnt exposure^25,31,40–42^. It is possible that these differences reflect more spatially restricted Wnt contact in our study compared to global Wnt exposure. It also seems likely that the bead attached to Wnt ligand in our study was so large as to prohibit endocytosis. Potentially related, recent evidence points to a role for the central region of APC in preventing clathrin-mediated endocytosis in the Wnt-off state^31^. Over time, the complexity of potential APC functions related to Wnt signaling has been gradually revealed. Previous work by our lab and others demonstrated that APC performs roles outside of its classically defined scaffolding function in the β-catenin destruction complex. For example, APC interaction with nuclear β-catenin leads to repression of Wnt target genes through several potential mechanisms: providing access to the transcriptional corepressor CtBP or the E3 ligase β-TrCP, β-catenin sequestration from transcriptional coactivator LEF-1/TCF, or facilitating β-catenin’s nuclear export^43–47^.

**Figure 8:**
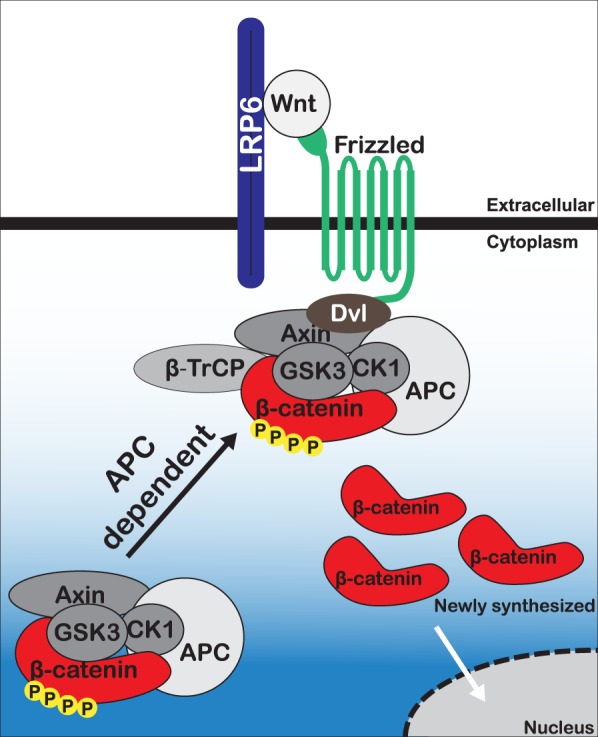
Proposed model of β-catenin destruction complex response to Wnt.

Together, our results suggest a novel mechanism of β-catenin destruction complex regulation by the APC protein (Fig. 8). In addition to its well-established role as a negative regulator of cytoplasmic β-catenin, we show that APC is also responsible for moving the β-catenin destruction complex to the cell membrane following Wnt exposure. We demonstrate for the first time that the endogenous β-catenin destruction complex reorients toward a localized Wnt signal in an APC-dependent manner in human colon epithelial cells of both normal and malignant origin. Finally, our data support a model whereby the destruction complex remains assembled and bound to β-catenin following Wnt ligand presentation. Because β-catenin accumulates in Wnt-treated cells, it appears that this β-catenin-bound complex is unable to effectively degrade β-catenin. Perhaps APC helps release modified β-catenin from the destruction complex, thereby enabling its proteasomal degradation as previously proposed^25^. Another possibility, is that Wnt stimulation leads to an APC-dependent membrane orientation of the destruction complex which results in complex inactivation. Upon removal of Wnt signal, this already assembled complex would be unlocked and able to process β-catenin for destruction. These two models are not mutually exclusive and might even be interdependent.

Future studies are necessary to clarify the precise mechanism of APC-regulated destruction complex trafficking to the membrane. Or study does not support an absolute requirement for Axin1 in Wnt-mediated destruction complex relocalization. Perhaps Axin2, a Wnt target, is able to compensate for Axin1 in this capacity. Axin has been postulated to localize to Wnt receptors following Wnt activation through a Dvl-dependent mechanism^48^. Based on the compromised destruction complex localization observed in DLD1 cells and APC-depleted HCEC 1CT cells, we suggest that APC is required for Axin docking to membrane-associated Dvl following Wnt exposure. In this light, our results strongly support the model proposed by Tacchelly-Benites *et al*., based on studies in *Drosophila*, that APC plays a major role in regulating Axin’s signalosome recruitment in response to Wnt signaling by facilitating phosphorylation of Axin by GSK-3β^49^. The compromised localization observed in DLD1 cells suggests that C-terminal domains contribute to this trafficking, potentially through cytoskeletal interactions^27–30,50,51^. The mechanism by which APC moves the β-catenin destruction complex toward the signalosome is a necessary focus for future studies in order to provide insight into colon epithelial cell biology and further elucidate unknown aspects of Wnt signal transduction. Overall, our findings provide additional mechanistic details of destruction complex behavior following Wnt exposure, and uncover a novel role for APC in Wnt signal transduction.

## Materials and Methods

### Cell culture and treatments

RKO, HCT116βm, DLD1, and RKO-APC^KO^ cells were cultured in DMEM (with L-Glutamine and 4.5 g/L Glucose; without Sodium Pyruvate) supplemented with 10% FBS and were maintained at 37 °C and 5% CO_2_. HCEC 1CT cells were generously provided by Dr. Jerry Shay, UT-Southwestern and cultured as described previously in X-media (4:1 DMEM/Medium 199) supplemented with EGF (20 ng/ml), hydrocortisone (1 μg/ml), insulin (10 μg/ml), apo-transferrin (2 μg/ml), sodium selenite (5 nM), and 2% cosmic calf serum^32^. Cells were maintained at 37 °C in low-oxygen incubators to reduce stress-induced senescence from normal atmospheric oxygen tension and to better recapitulate physiological conditions, as described previously^52^. For immunofluorescence assays, cells were seeded onto fibronectin/Poly-D-Lysine coated coverglass (Neuvitro) at 25% confluence and incubated at least 24 hrs prior to treatment with 5 μl beads/well for 12–14 hrs. HCEC 1CT cells were seeded at 10–15% confluence prior to siRNA transfection and Wnt-bead treatment.

For siRNA-mediated inhibition, HCEC 1CT cells were transfected using Lipofectamine 3000 (Invitrogen) according to the manufacturer’s instructions with 37.5 nM of each siRNA targeting human APC (Smartpool siRNAs 1–3; Dharmacon), human Axin1 (Smartpool siRNA 4; Dharmacon) or nontargeting siControl siRNA (Dharmacon). Cell media was changed one day following siRNA transfection, and cells were grown 48 hrs prior to Wnt-bead or Unloaded-bead treatment.

### Immobilization of Wnt protein

Wnt3a was immobilized onto Dynabeads as described previously^39^. Briefly, 2.8 μm Dynabeads M-270 Carboxylic Acid (Invitrogen) were activated by NHS/EDC (Sigma, 50 mg/ml each in cold 25 mM MES pH 5) then washed three times with cold MES buffer. Wnt immobilization was performed by diluting 0.5 μg of purified Wnt3a protein (Peprotech, #315-20) in cold MES buffer and incubated at room temperature (RT) for 1 hr. To quench non-reactive carboxylic acid groups, beads were incubated with 50 mM Tris pH 7.4 at RT for 15 min. Beads were washed twice in PBS pH 7.4 before final resuspension in 400 μl PBS/0.5% BSA and stored at 4 °C. Unloaded-beads were prepared in parallel by incubating 1 hr in MES without Wnt. Wnt3a activity following bead immobilization was verified using a TOPflash luciferase reporter assay^53^.

### Immunofluorescence

Cells were briefly rinsed in PBS prior to fixation. Cells were fixed in 4% PFA in Brinkley’s Buffer 1980 (80 mM PIPES pH 6.8, 1 mM MgCl_2_, 1 mM EGTA) for 20 min at RT, washed two times in PBS prior to permeabilization in TBS/0.2% Triton X-100 for 5 min. Cells were washed in TBS two times prior to incubation for 1 hr at RT in blocking buffer containing TBS/0.2% Triton X-100, 1% BSA, and 3% Normal Goat Serum. Primary and secondary antibodies were incubated for 1 hr at RT. Cells were washed in TBS three times following primary and secondary antibody incubations. Coverslips were mounted and counterstained with Prolong Diamond Antifade Mountant with DAPI (Invitrogen). Staining of FZD7 was performed without cell permeabilization or use of detergent to keep the cell membrane intact. Nuclei were counterstained with 5 μg/ml Hoechst 33342 (Invitrogen) in PBS for 5–10 min prior to mounting with Prolong Antifade (Invitrogen). Antibodies were diluted in blocking buffer as follows: anti-APC-M2 rabbit pAb (1:4,000^54^), anti-Axin1 rabbit mAb (1:500, Cell Signaling, #2087), anti-β-catenin mouse mAb (1:250, BD Transduction Laboratories, #610154), anti-CK1α rabbit pAb (1:500, Bethyl Laboratories, #A301–991A), anti-GSK3β rabbit pAb (1:500, Bethyl Laboratories, #A302-049A), anti-β-TrCP rabbit pAb (1:500, Abcam, #71753), and anti-Frizzled-7 rabbit pAb (1:500, EMD Millipore, #06-1063), Alexa Fluor 488 and 568 conjugated secondary antibodies (1:1,000, Invitrogen). Stained cells were examined using an Axioplan microscope (Zeiss) with a X100 objective. Images of stained cells were captured using an Orca R^2^ digital camera (Hamamatsu).

### Immunoblotting

Cells were washed 1x in PBS prior to harvesting in pre-heated high-salt sample lysis buffer (20% glycerol, 2% SDS, 30% 10x PBS, 2.5% β-mercaptoethanol). Scraped cells were transferred to Eppendorf tubes, heated at 95 °C for 1 min, pulled through an insulin syringe three times, and heated again. Samples were separated on 7.5% SDS-PAGE (Bio-Rad, TGX FastCast Acrylamide Kit) using Tris-Glycine running buffer and transferred to a nitrocellulose membrane (GE) with a 0.45-μm pore size. Antibodies were diluted in Odyssey Blocking Buffer TBS (LI-COR) as follows: anti-APC-M2 rabbit pAb (1:2,000), anti-β-catenin mouse mAb (1:1,000), anti-Axin1 goat (1:500, R&D Systems #AF3287), and anti-β-actin mouse mAb (Sigma) (1:1,000), IRDye 680LT and 800CW anti-rabbit or anti-mouse secondary antibodies (1:15,000). Immunoblots were imaged on a LI-COR Odyssey CLx imaging system.

### Wnt-bead pull-down

Cells were grown in 6-well tissue culture plates and treated with 40 μl Unloaded-beads or Wnt-beads for four hours. Following bead treatment, cells were briefly washed in 1x PBS prior to lysis in 200 μl lysis buffer, described previously (150 mM NaCl, 30 mM Tris pH 7.5, 1 mM EDTA, 1% Triton X-100, 10% glycerol, 0.1 mM PMSF, 0.5 mM DTT, and HALT protease and phosphatase inhibitors)^25^. After addition of lysis buffer, cells were scraped into 1.5 ml tubes and rotated at 4 °C for 30 min. Beads were isolated using a magnet and the supernatant was transferred to a new tube. Beads were washed three times in 500 μl of cold lysis buffer. Following the final wash, beads were resuspended in 40 μl cold PBS and 20 μl 3X SDS sample buffer. Samples were analyzed by western blot as described above.

### Line scan analysis

ImageJ/FIJI (National Institutes of Health, Bethesda, MD) was used for line scan analyses to quantify protein localization in relation to a Wnt- or Unloaded-bead. Line width was set to 50, corresponding to approximately the size of a Dynabead. Lines were drawn beginning at the bead and across the cell, moving through and across the center of the nucleus. For graphical presentation in (Fig. 1c–e), each scan was set to zero by subtracting the lowest intensity value across the line. At least five representative cells were measured per condition. Refer to Supplemental Figs. S1–S3 for line scan analysis of RKO, HCT116βm, and DLD1 cells.

### Statistical analysis

Blinded scoring was performed on a subset of samples using the scoring system presented in Fig. 1a. Slides were covered and numbered prior to scoring and imaging, and decoded following completion of each experiment. Experiments were performed a minimum of three times with at least 25 cells scored per condition per experiment. T-tests (two-tailed) were performed using GraphPad Prism 8 to determine statistical significance between score frequency in Wnt-bead and Unloaded-bead groups as well as between control siRNA and APC siRNA samples in the HCEC 1CT experiments.

## Supplementary information

Supplementary figures and legends.
