# Supplementary material for: APC controls Wnt-induced β-catenin destruction complex recruitment in human colonocytes
Source: Sci Rep. 2020 Feb 19;10:2957. doi: 10.1038/s41598-020-59899-z (PMC7031393; doi:10.1038/s41598-020-59899-z)
Supplement: Supplementary file 1 — Supplementary figures and legends. [file 41598_2020_59899_MOESM1_ESM.pdf]

APC controls Wnt-induced β-catenin destruction complex recruitment in human colonocytes

Taybor W. Parker and Kristi L. Neufeld[1]

Department of Molecular Biosciences, University of Kansas, Lawrence, KS

[1]Corresponding author: Kristi L. Neufeld, 7049 Haworth Hall, 1200 Sunnyside Ave., University of Kansas, Lawrence, KS 66045; phone (785)864-5079; email: klneuf@ku.edu

Author Information:
Taybor W. Parker, parkertw@ku.edu, ORCID: 0000-0002-3829-7554
Kristi L. Neufeld, klneuf@ku.edu, ORCID: 0000-0003-3653-9385

Supplemental Figure Legends

Supplemental Figure 1. **Line scan analysis of signalosome and destruction complex components in RKO cells.** Line scans were performed on RKO cells containing an intact Wnt signaling pathway using ImageJ/FIJI (NIH). Lines were drawn beginning at the Wnt- or Unloaded-bead and run across the cell nucleus. Line colors correspond to bright-field (black), DAPI (blue), and protein (red). If brightfield image was unavailable, the black line corresponds to DAPI. Line scans correspond to A) APC, B) Axin1, C) β-catenin, D) CK1-α, E) GSK-3β, F) β-TrCP, and G) FZD7. Line scans were performed on five cells per condition.

Supplemental Figure 2. **Line scan analysis of signalosome and destruction complex in HCT116βm cells.** Line scans were performed on HCT116βm cells containing stabilized β-catenin through Ser45 deletion. Lines were drawn beginning at the Wnt- or Unloaded-bead and run across the cell nucleus. Line colors correspond to bright-field (black), DAPI (blue), and protein (red). If brightfield image was unavailable, the black line corresponds to DAPI. Line scans correspond to A) APC, B) Axin1, C) β-catenin, D) CK1-α, E) GSK-3β, F) β-TrCP, and G) FZD7. Line scans were performed on five cells per condition.

Supplemental Figure 3. **Line scan analysis of signalosome and destruction complex in DLD1 cells.** Line scans were performed on DLD1 cells which harbor truncated APC. Lines were drawn beginning at the Wnt- or Unloaded-bead and run across the cell nucleus. Line colors correspond to bright-field (black), DAPI (blue), and protein (red). If brightfield image was unavailable, the black line corresponds to DAPI. Line scans correspond to A) APC, B) Axin1, C) β-catenin, D) CK1-α, E) GSK-3β, F) β-TrCP, and G) FZD7. Line scans were performed on five cells per condition.

Supplemental Figure 4. **HCEC 1CT images from Figure 7.** Immunofluorescent microscopy images of HCEC 1CT cells following APC, Axin1, or control siRNA treatment and Wnt-bead treatment. Images shown are individual channels of the images used in Figure 7. Immunofluorescent images of (A) APC and β-catenin, (B) Axin1, and (C) GSK-3β following siCtl and siAPC treatment. (D) Immunofluorescent images of APC and β-catenin following siAxin1 treatment.

Supplemental Figure 5. **Full-length western blots.** (A) Wnt-bead “pull down” assay demonstrated in Figure 3. (B) APC knock-down shown in Figure 7. (C) Axin1 knock-down shown in Figure 7. Each individual set of images are taken from the same gel/membrane.

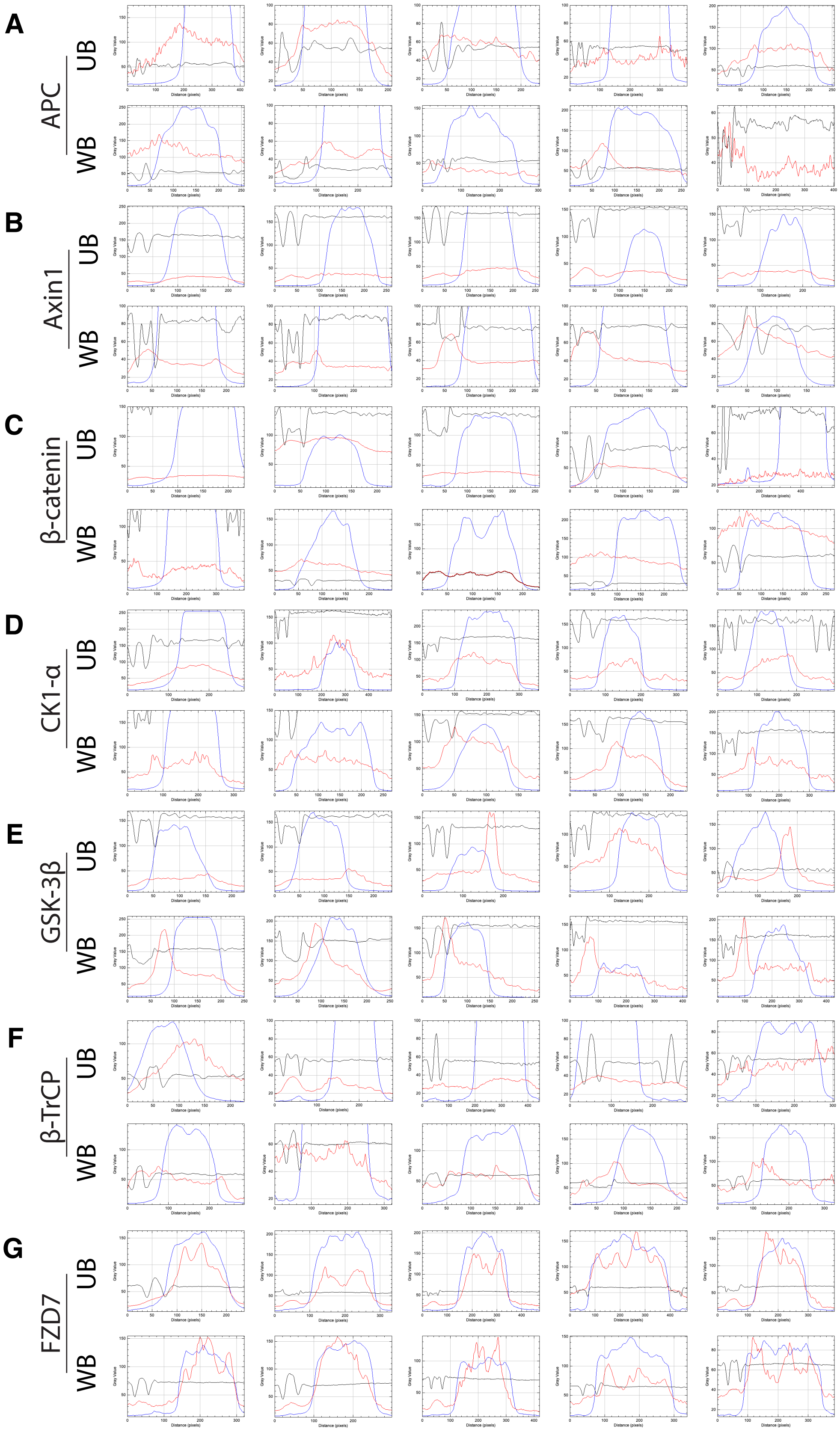

Supplemental Fig. 1

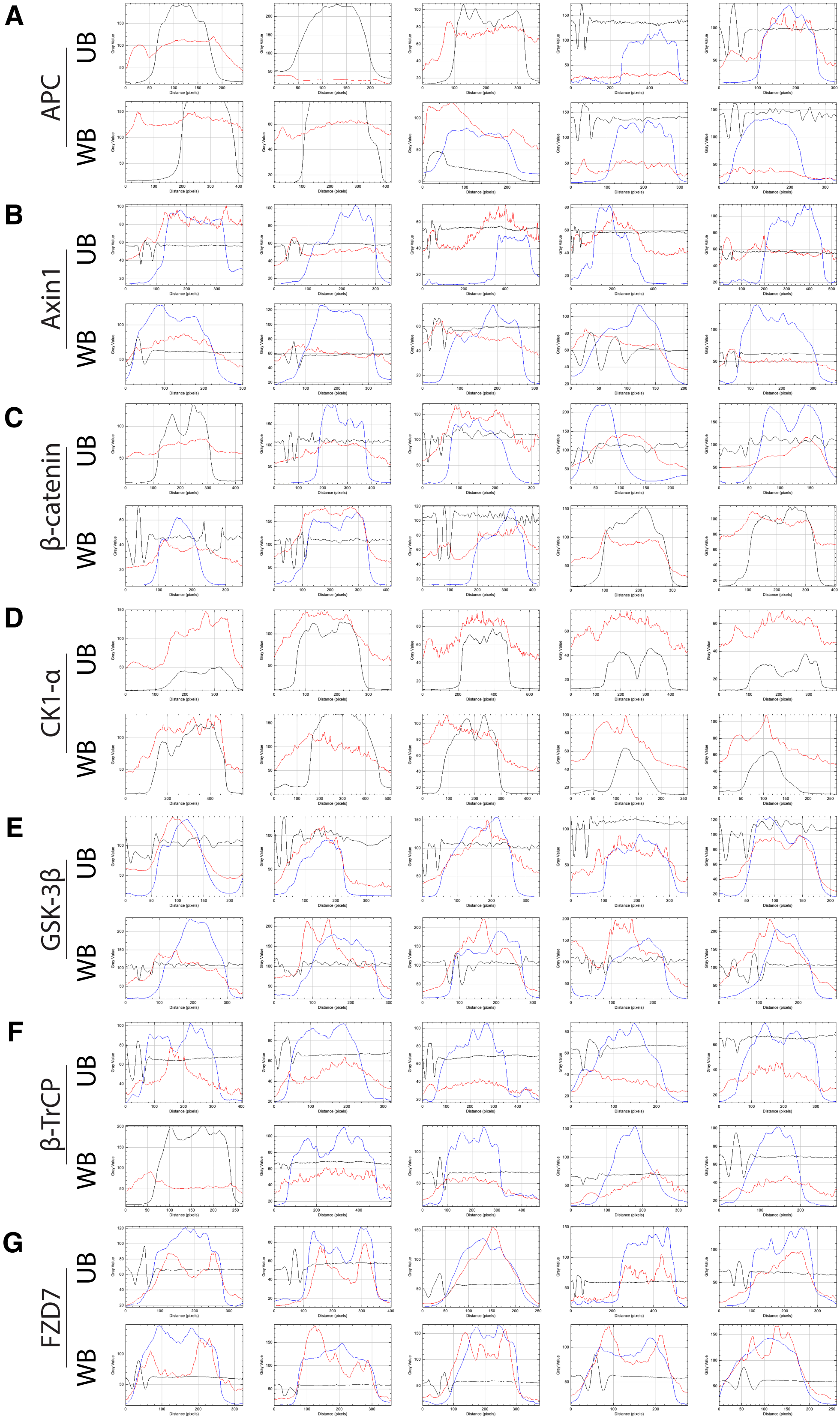

Supplemental Fig. 2

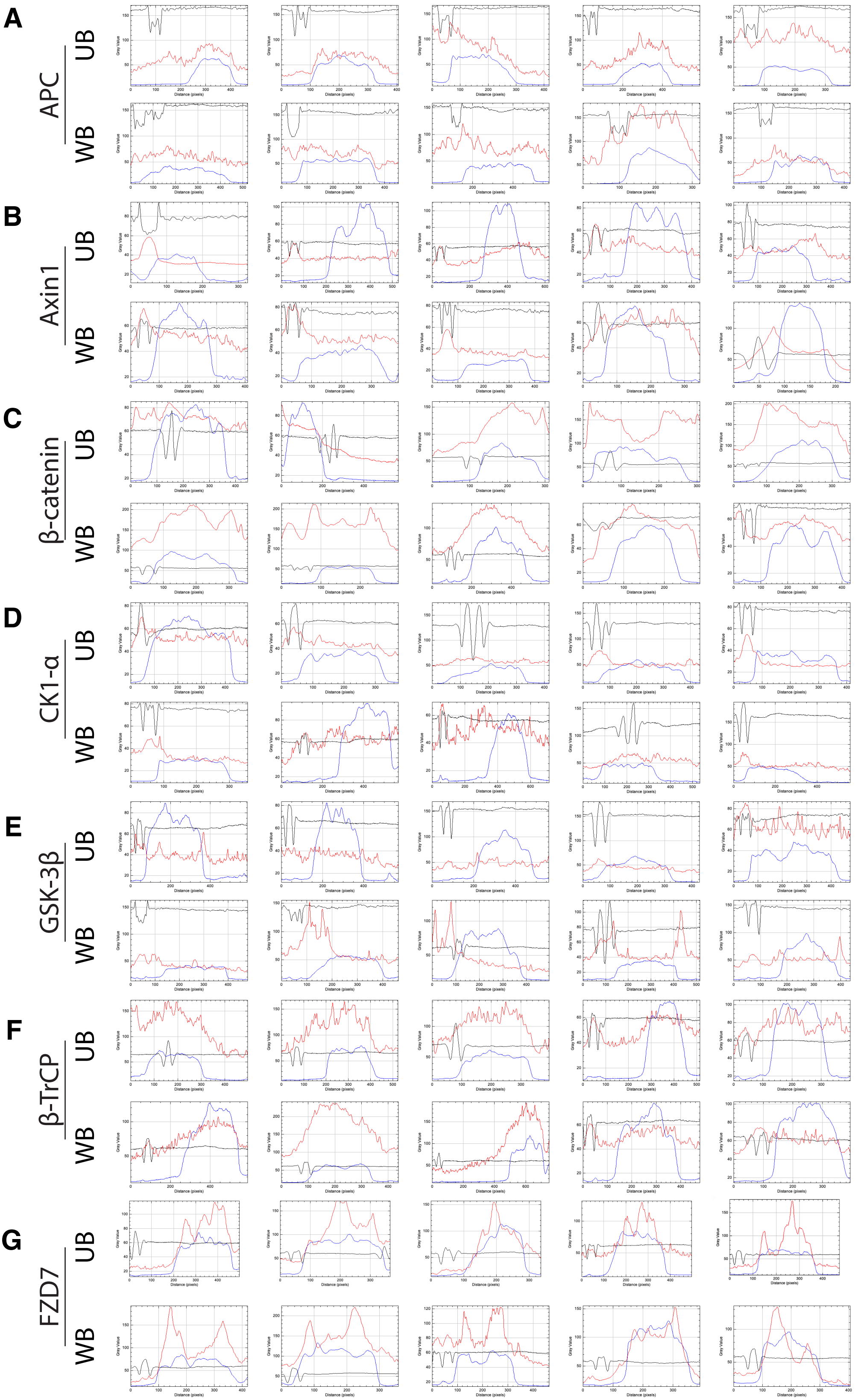

Supplemental Fig. 3

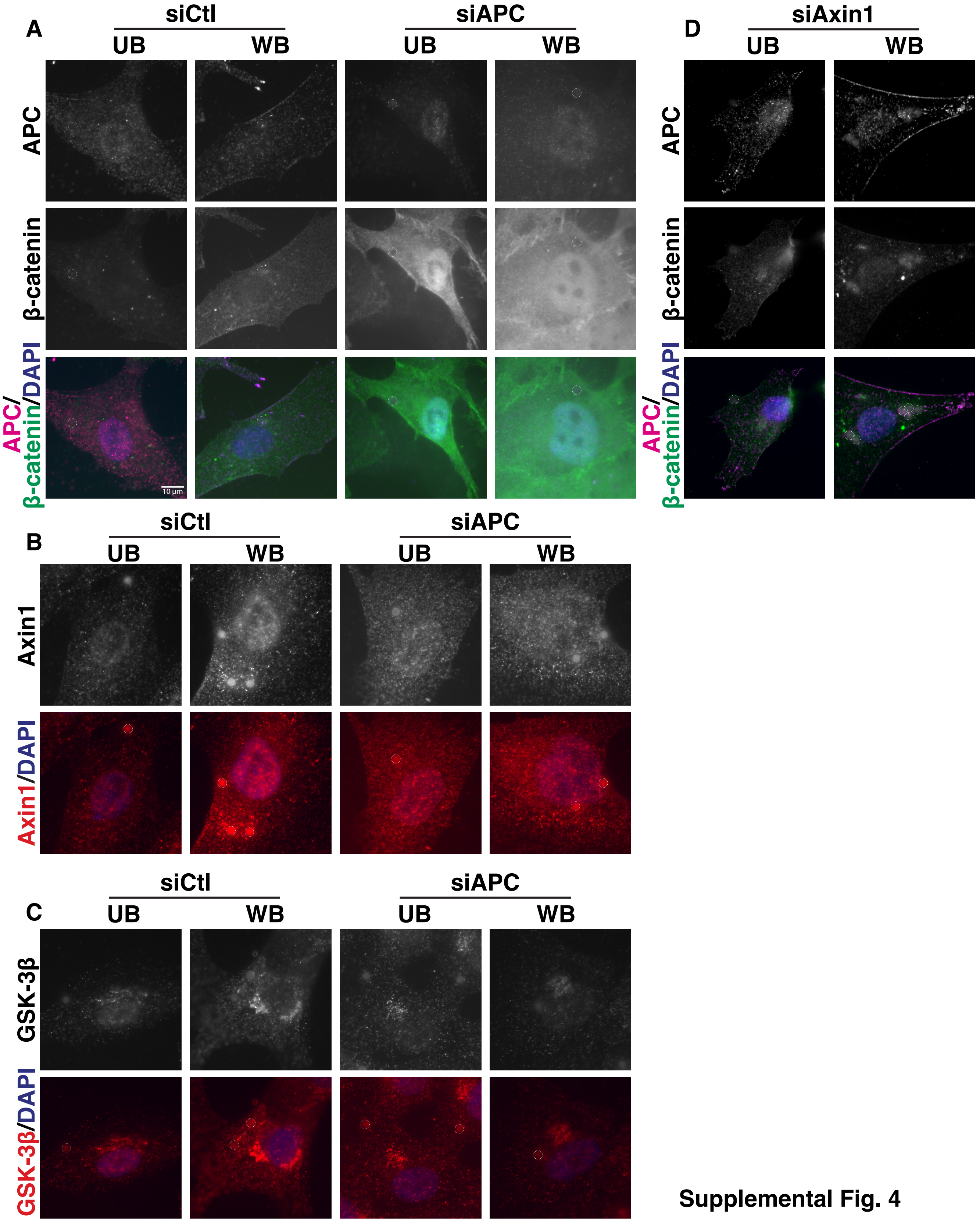

Supplemental Fig. 4

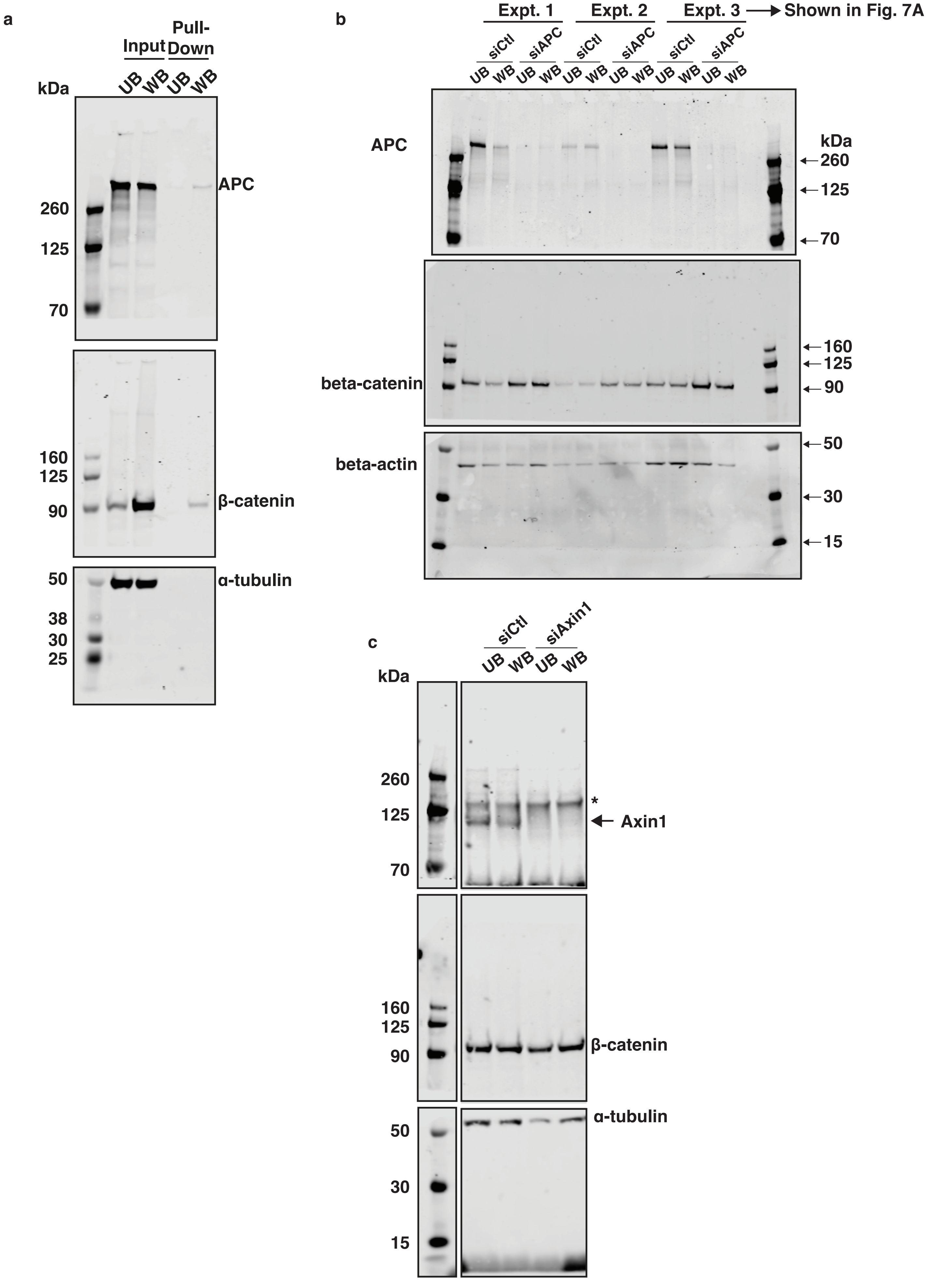

Supplemental Fig. 5
